# Transforming the wellbeing focus in education: A document analysis of policy in Aotearoa New Zealand

**DOI:** 10.1080/17482631.2021.1879370

**Published:** 2021-02-02

**Authors:** Joanna Higgins, Suskya Goodall

**Affiliations:** Faculty of Education, Victoria University of Wellington l Te Herenga Waka, Wellington, New Zealand

**Keywords:** Education policy, wellbeing, ecological systems, strengths-based, document analysis

## Abstract

**Purpose**: Understanding the nature of wellbeing as multidimensional and complex provides a policy window to generate a strengths-based policy orientation to promote wellbeing in education settings. The purpose of this exploratory paper is to map how wellbeing is interpreted across public education policy documents in Aotearoa New Zealand.

**Method**: To explore the narrative that this group of documents weave, we draw on models of holistic wellbeing, ecological systems and appreciative inquiry. Policy documents were analysed using text mining software to track notions of wellbeing; their occurrence and co-occurrence with related concepts.

**Results**: Key findings include the predominance of wellbeing, the interrelatedness of wellbeing with relationships, and the predominance of student wellbeing over the wellbeing of other stakeholders, highlighting that current education policy does not interpret wellbeing as relational, complex or contextual.

**Conclusion**: We argue that interpreting such documents through a wellbeing lens demonstrates the complexity and disparity of the conceptualization and contextualization. We assert that it is critical to explore possibilities for deliberate and ecological wellbeing connections within educational policy and practice for the good of all stakeholders.

## Introduction

There is increasing urgency to prioritize wellbeing in public policy across international jurisdictions. This appears to have created a policy window to increase the wellbeing focus in public policy, including that impacting education. The term “policy window” points to the temporal dimension of policy formation, and to identifying the catalytic moment for policy change (Kingdon, 2003, p. 165). In comparative education policy research, Steiner-Khamsi (2006) also referred to “a window of opportunity” and linked this to the phenomenon of “cross-national policy borrowing” (p. 670). In relation to education policy, the international body, the United Nations Educational, Scientific and Cultural Organization (UNESCO), states that “solid, coherent policies and plans are the bedrock on which to build sustainable education systems, achieve educational development goals and contribute effectively to lifelong learning” (n.d.).

Prioritizing wellbeing within this emergent policy window is particularly urgent given the advent of the global coronavirus disease (COVID-19) pandemic and the associated government responses and public policy development. This article explores the coherence of wellbeing constructs in educational policy and addresses the question: How can public policy present wellbeing so that the interrelatedness, complexity, and contextual nature of wellbeing is reflected in educational settings? Our aim is to re-examine ways of reading Aotearoa New Zealand policy documents using a strengths-based lens to find possibilities for transformative practices that promote wellbeing in education. The examination of education policy within the bicultural context of Aotearoa New Zealand that arises out of the political imperative under the obligations of Te Tiriti o Waitangi (The Treaty of Waitangi) partnership and calls for both Māori (indigenous peoples) and non-Māori knowledge to inform policy (Durie, 2011). While biculturalism is clearly a strength when it comes to wellbeing in educational policy, it also may generate appropriation of Māori models in which the cultural essence is threatened by a simplification for policy purposes of the multifaceted notions of wellbeing (McKinley, 2005).

## Policy framing

Reflecting political priorities of government organizations, policy can guide action or be seen as a statement of intent (Ball, 2015). Policy formation or policy drivers by definition are generally prompted by a political response to a problem or issue. However, one of the key questions in public policy studies is when or under what circumstances there is receptiveness towards new or reform ideas (Howlett & Ramesh, 2003; Kingdon, 2003; Sabatier & Jenkins-Smith, 1993). We suggest that this catalytic moment of international and local issues of wellbeing has generated a renewed societal interest, and associated political responses, in ways that individuals and systems might enhance wellbeing for all. There is mounting evidence that societal wellbeing, heightened at this moment in history, is at a crisis point with statistics showing rates of poor mental health and suicide increasing for Western English-speaking countries. Therefore, a challenge for education policy is how to use a strengths-based frame that promotes the notion of wellbeing for all, rather than ad hoc policy development in response to an issue that is part of a crisis of wellbeing in society.

Strengths-based social policy can be contrasted with policy prompted by an issue (Rapp et al., 2006). A strengths-based approach to social policy development can be traced back to Chapin (1995). Interest in this perspective has continued over the last two decades with various scholars using strengths-based approaches to examine social policy (Hill, 2008; Maton et al., 2004; Rapp et al., 2006). In relation to problem-based policy, Rapp et al. noted the potential for associated pathologising and a focus on assessment or gathering evidence to measure the policy’s success. We suggest that education policy formulated to address a problem, at times referred to as evidence-based policy, follows a similar pattern of a response to an issue and targeted assessments to measure a policy’s success. In contrast, a strengths-based framing is claimed to be a more inclusive approach to policy formulation and to offer “empowering policy options” (Rapp et al., 2006, p. 4). Furthermore, just as Hill (2008) argued for social work, education embodies professional principles, ethics and values that are foundational for a strengths-based practice. We argue for a shift to a strengths-based lens to avoid policy development associated with wellbeing being “a piecemeal of ‘fixing problems’” rather than “generating forms of institutional transformation and regeneration” (Ball, 2015, p. 309).

Policy, as argued by Ball (1993, 2015), is underpinned by two contrasting conceptualizations: policy as discourse and policy as text. From a historical perspective, policy can be viewed as a discourse reflecting social and economic influences and ideas over time (Hard et al., 2018; Ozga, 2000). Ball (1993) stated, “Discourses are what can be said, and thought, but also who can speak, when, where and with what authority” (p. 14). The notion of power is central to this Foucauldian view of policy, not only which discourses are constructed and how they change, but also their influence on daily life (Ball, 2015). In his 2015 reflections on his 1993 paper, Ball (2015) observed that policy research to date had predominantly focused on policy as text rather than policy as discourse: “a lot more focus on what is written and said, rather than how those statements are formed and made possible” (p. 311). The focus in this article is on policy as text or what is written through the “analysis of key or recurrent words or phrases in policy documents” (p. 311). We are, as Ball (2015) suggested, staying on “the surface of things, taking policy at face value and re-inscribing its claims to coherence in our analyses (p. 311). We suggest that this can be a useful initial approach for identifying apparent omissions and multidimensionalities across recent policy documents that incorporate renewed attention on wellbeing in educational settings.

Our aim is to re-examine ways of reading Aotearoa New Zealand policy documents using a strengths-based lens to find possibilities for transformative practices that promote wellbeing in education. The examination of education policy within the bicultural context of Aotearoa New Zealand that arises out of the political imperative under the obligations of Te Tiriti o Waitangi (The Treaty of Waitangi) partnership, and calls for both Māori (indigenous peoples) and non-Māori knowledge to inform policy (Durie, 2011). While biculturalism is clearly a strength when it comes to wellbeing in educational policy, it also may generate appropriation of Māori models in which the cultural essence is threatened by a simplification for policy purposes of the multifaceted notions of wellbeing (McKinley, 2005).

## Wellbeing as a construct

In public and political arenas, with increasing popularity, wellbeing features as a ubiquitous term. The United Nations (UN), the World Health Organization (WHO) and the Organization for Economic Co-operation and Development (OECD) have been particularly influential in the focus on wellbeing within global policy. As a framework for country action, *Health in All Policies* (2014) was produced by the WHO to ensure health as societal goal underpins policy formation. In 2015, the UN envisioned a world where there is an assurance of wellbeing, physically, mentally and socially. Combined, the vision and focus of these organizations heightened global awareness of wellbeing across OECD countries and sectors. The UN Sustainable Development Goals (2015) were pivotal to an underpinning of wellbeing within public policy. The third sustainable development goal of *Good Health and Well-being* is “ensuring healthy lives and promoting the well-being for all at all ages is essential to sustainable development” (p. 20). Within an educational context, the fourth sustainable development goal of *Quality Education* stated that “obtaining a quality education is the foundation to improving people’s lives and sustainable development”. Enacting the fourth goal, the OECD (2018) launched a new project called *The Future of Education and Skills 2030*. The foundations and transformative competencies of the project include physical, mental, social and emotional elements of wellbeing (2018). In 2019, the WHO coordinated multiple global health and development organizations to collaboratively expedite progress of global health goals through its Global Action Plan for Healthy Lives and Well-being for All (WHO, 2019).

The burgeoning use of the term “wellbeing” in popular culture, politics and academic research denotes movement away from relying on the GDP of a nation as a measure of wellbeing. Despite the global attention, wellbeing remains slippery as a construct and elusive to define (Ereaut & Whiting, 2008; Pollard & Lee, 2003). This may have prompted theorists investigating wellbeing to elect divergent pathways in seeking to define wellbeing. Some researchers look to the notion of psychological wellbeing encompassing subjective wellbeing, engagement, relationships, mastery, life purpose, optimism and autonomy (Deci & Ryan, 2008). Simplifying these elements of psychological wellbeing, various theorists perceive wellbeing as a combination of “feeling good” and “functioning effectively” (F. A. Huppert & Johnson, 2010; F. Huppert, 2009; Waters, 2011). Taking “a less is more” approach, Dodge et al. (2012) describe wellbeing as a point of balance influenced by “an individual’s resource pool” and the challenges they face (p. 213).

Alternatively, in an effort to include external wellbeing factors, La Placa et al. (2013) inclusively consider the wellbeing of “family, community and society as a whole” (p. 116). This perspective is conceptualized by those who construe wellbeing ecologically by “holistically taking into account all effective factors in all possible domains” (Paterson & Grantham, 2016, p. 92). Theorists converge on several characteristics of wellbeing: it is a construct that is multidimensional, complex, and based on “good”. Thus, for the purpose of this piece, we draw on McCallum and Price (2016) definition of wellbeing because it encapsulates its multidimensional and relational complexity as follows:

Wellbeing is diverse and fluid respecting individual, family and community beliefs, values, experiences, culture, opportunities and contexts across time and change. It is something we all aim for, underpinned by positive notions, yet is unique to each of us and provides us with a sense of who we are which needs to be respected. (p. 17)

In conceptualizing wellbeing, it is worth explaining several interrelated terms that exist in the literature. The terms “wellness” or “welfare” are synonymous with “wellbeing” (Brasfield, 2015; Pfieffer, 2017), whereas the term “flourishing” is associated with optimal feeling and functioning (Hone et al., 2014; Huppert & So, 2013). In seeking to explore the construct of flourishing, Diener et al. (2010) contribute criteria of distinguishing characteristics including meaning, purpose, positive relationships, competence, engagement, self-esteem, respect, optimism, and contribution to others’ wellbeing. Based on their large-scale European study of flourishing citizenry, F. Huppert and So (2013) added vitality, resilience and self-determination to Diener’s criteria.

Moving to the bicultural policy setting of Aotearoa New Zealand, Māori models demonstrate a holistic framing of wellbeing. A Māori model of hauora (holistic wellbeing), known as Te Whare Tapa Whā, (Durie, 1994) provides a holistic lens and multidimensional conceptualization of wellbeing in this paper. Adopted within New Zealand health and education sectors, the model is a house with four walls, each representing a dimension of hauora: taha tinana (physical), taha hinengaro (mental and emotional), taha whānau (social) and taha wairua (spiritual). Each interdependent dimension or wall requires development to metaphorically hold up the roof. Embedded within Durie’s original model was the foundational notion of whenua (land) as place and context. However, the version used in the education sector was void of this element. Although there are ongoing debates about the appropriation of such models in educational settings (McKinley, 2005), the strength of Te Whare Tapa Whā is as a multidimensional construct of wellbeing that is accessible and has potential for practical application. The elements of social, emotional, mental and spiritual wellbeing indicate what it means to be well, beyond the physical realm. The multidimensional and relational complexity of wellbeing connects to Te Whare Tapa Whā and is invoked through our chosen definition (McCallum & Price, 2016) to incorporate a holistic, and potentially ecological, lens.

## Methodology

An ecological framing aligns with a strengths-based orientation to policy (Weaver-Hightower, 2008). Conceptualizing education policy research through an ecological metaphor, Weaver-Hightower recognizes the complexity and messiness of the policy process. Taking each policy as an ecological system with multiple layers or strata (Bronfenbrenner, 1979) that is “complex, interdependent, and intensely political” (Weaver-Hightower, p. 154) highlights the multiple dynamics arising from stakeholder collaboration for a policy issue, such as wellbeing. Another methodological influence is Appreciative Inquiry, which provides a strengths-based approach to policy analysis, seeking explanations and possibilities to explore in maximizing potential. Developed for the business sector by David Cooperrider in 1986, Appreciative Inquiry is a philosophy that focuses on potential, involvement and cooperation to create positive sustainable thinking and change. Waters and White (2015) described Appreciative Inquiry as holistic and collaborative methodology and noted how it can be adopted to frame wellbeing policy initiatives. In what follows, we use an ecological and appreciative inquiry framing to interpret the potential of 15 wellbeing-related policies impacting education.

Following our focus on policy as text (Ball, 2015), this paper employs document analysis as the principal method of inquiry. Document analysis provides a specialized form of qualitative research. Despite some theorists warning against over-reliance on documents (Bowen, 2009), others maintain that documents are a ubiquitous yet largely unnoticed part of our everyday lives, requiring more of a systematic analytic focus across research traditions (Rapley & Jenkings, 2010). Olson (2010) presents the notion of documents, including policy, as records of human activity, which provide a data source that is both important and useful. Rapley and Jenkings propose several approaches to document analysis within educational contexts, one of which is meta-synthesis. This approach focuses on the content of documents with an analytic intertextual purpose. We contend that the analysis of documents can reveal meaning, contribute understanding and illuminate insights relevant to particular research problems, which aligns to this paper’s purpose.

Content analysis and thematic analysis often feature as practical elements of document analysis. Processes used with documents vary depending on purpose and context, however, Bowen (2009) identifies key elements often used. “The analytic procedure entails finding, selecting, appraising (making sense of), and synthesising data contained in documents” (p. 28). In addition to document analysis, we have selected a narrative focus to highlight wellbeing within the documents.

In exploring the idea of the positioning of documents in research, Prior (2008) provided a useful perspective on the role of documents in social research that is applicable to this analysis of wellbeing policy. Hence, we follow his approach of re-positioning documentation through “treating documents as informants” that “builds up an idea of being ‘actors’ [albeit inanimate] in their own right” (p. 822). Rather than a focus on content, Prior (2008) explains a focus on use and function to explicate the ways documents function and impact on schema, in our case that of system-level wellbeing in education. The scope of our analysis does not extend to investigating the origins of the documents.

The documents analysed are relevant to a range of stakeholders, who can be considered characters or actants within the education system; for instance, teachers, school leaders, students, community. To tell the story of our document analysis we present a policy narrative (Prior et al., 2012). Prior (2014) explained the useful association between narrative and policy discourse, delving into a process of narrative policy analysis. We have adopted this narrative form to relate how wellbeing emerges in the policy documents analysed, however, we consider policy as text, rather than discourse (Ball, 2015). To complement ecological and appreciative frames, we embraced narrative as a research method to analyse and discuss how the story of wellbeing was told across these documents.

## Method

The document analysis of 15 texts containing thousands of words and concepts called for the “deployment of complex algorithms and text-mining procedures” (Prior, 2014, p. 15). As researched by Prior, selection of IBM SPSS Modeller (2018) was based on its powerful algorithms and analytical tools to enable insights across the full data set in relation to wellbeing. Text mining algorithms applied rules of selection and analysis to map comprehensive relationships and associations between wellbeing constructs. Mapping the interrelatedness of concepts informed the policy narrative. To identify the most important concepts occurring across the documents, global word frequency weightings were calculated. Delving deeper, associations between concepts were mapped to show their co-occurrence; of particular interest were the similarity and syntactical interrelationships. Specific technical terms are further unpacked in the following section.

Fifteen documents met the following six inclusion criteria:

- generated in Aotearoa New Zealand
- intended for use in the early childhood, primary and secondary education sectors
- released within the last twenty years
- has legal status and/or referenced to an official document
- referenced the construct of wellbeing, and
- was representative of the policy functions.

These functions included curricula, leadership guidelines, system evaluation and improvement, legal and regulatory frameworks and professional standards. This is not to suggest that all policy documents which have reference to the construct of wellbeing were included in the analysis. In performing this analysis, we firstly acknowledge the diverse time periods, purposes and organizational influences across the collection, and the purposeful mindfulness of these factors for the duration of the process.

## Analysis and results

**Figure 1.**
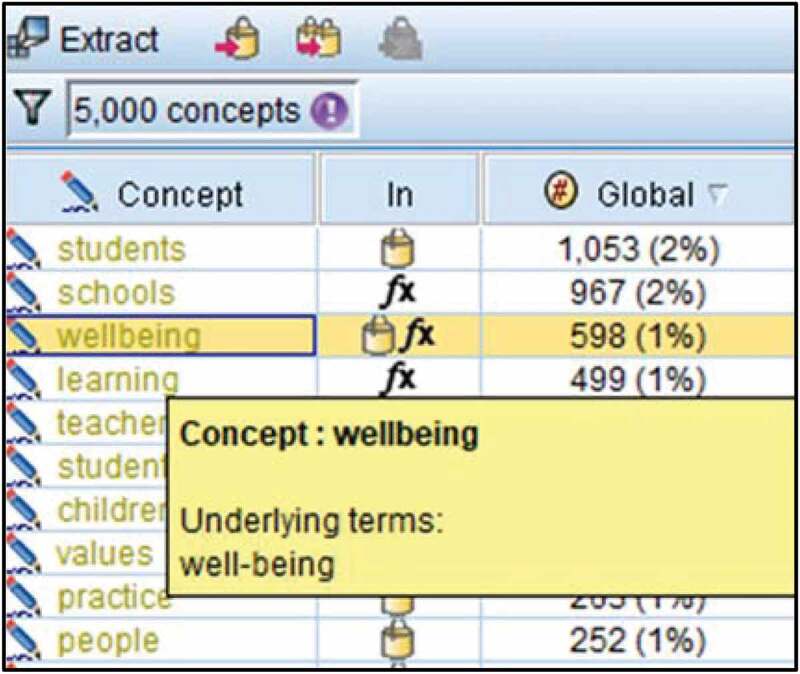
Word frequency weightings across the fifteen documents

In this section, the process and findings have been purposefully interwoven to form a narrative analysis of wellbeing across documents. Initially, concepts were identified from word frequency weightings inclusive of elementary word associations following Prior’s (2014) content analysis that distinguish the most important concepts across the documents. Of the 5000 concepts identified from word frequency weightings, wellbeing was the third most frequently mentioned global concept after “students” and “schools”. “Well-being” was an underlying term within the concept of “wellbeing”, as shown below within the ten predominant global concepts in Figure 1.

**Figure 2.**
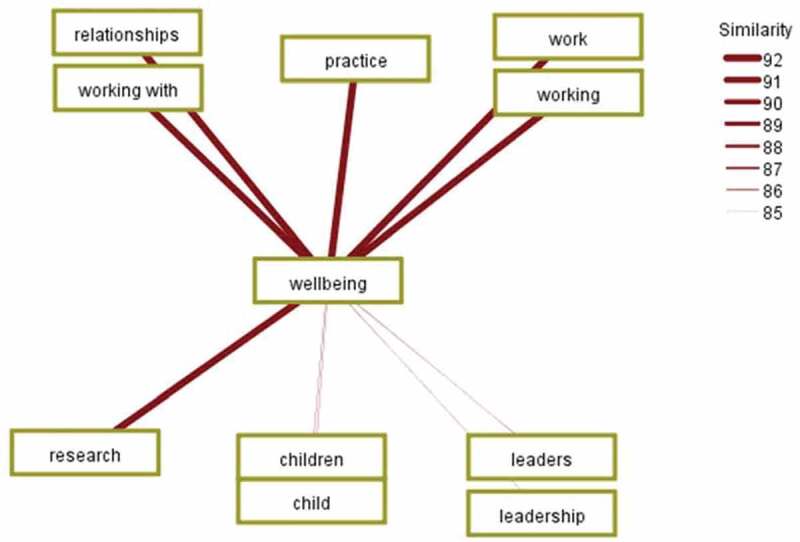
Co-occurrence of wellbeing with the strongest ten concepts

Secondly, the interrelatedness of “wellbeing” with other concepts was calculated to reveal the highest levels of co-occurrence with the concepts of “practice”, “relationships”, “work” and “research”. The co-occurrences were generated using a similarity metric, which calculated relative link strength based on the frequency that the two concepts appear apart simultaneously with how often the two concepts appear together. “Wellbeing” is mapped on a concept cluster to show the top ten co-occurrent links. When a pair of concepts tend to appear more often together than apart, they are assigned a higher strength value and a thicker line, as displayed in Figure 2 below. Like concepts are displayed in proximity for visual clarity. In some cases, this has meant that some lines pass behind another concept box.

**Figure 3.**
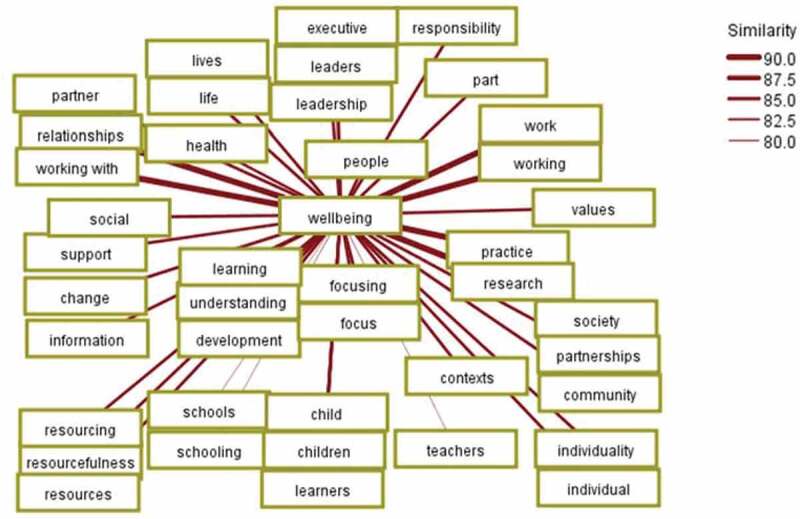
Co-occurrence of wellbeing with forty-one conceptual connections

In order to delve into the complexities, the concept mapping was expanded to show the co-occurrence for a larger number of concepts. The analysis found a notable variation in the wellbeing of different groups of people. The first ten co-occurrences with a similarity metric featured both “children” and “leaders”, as displayed in Figure 2 above. However, “community” featured at the twentieth, “society” at the thirty-fifth, “schools” at thirty-ninth and “teachers” at forty-first, the latter of which is depicted in Figure 3 below. Once again, like concepts are clustered for visual clarity.

**Figure 4.**
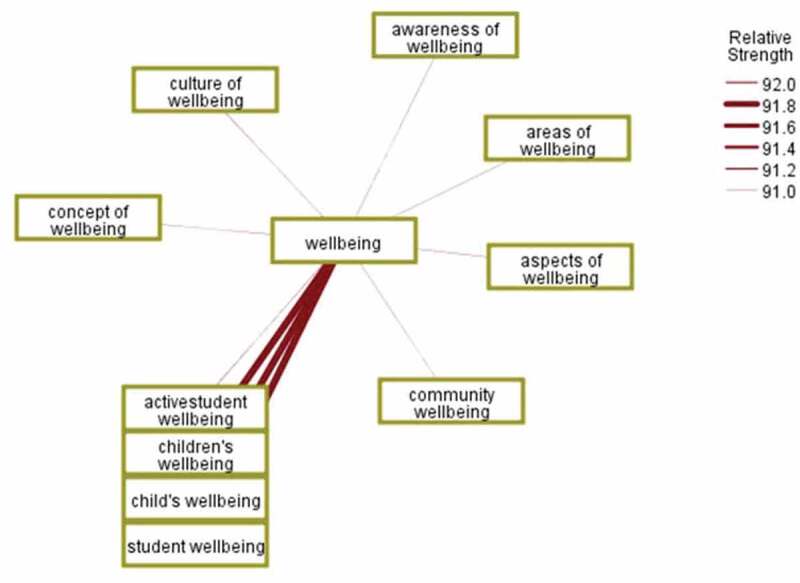
Syntactical occurrence of “wellbeing” with the strongest ten concepts

To unpack nuances of wellbeing across the documents, the concept links that were not co-occurring, were found using a confidence metric for syntactical interrelatedness. “Wellbeing” related to “children” or “students” had the strongest relationship according to the confidence metric. It is interesting to note, the purpose of the confidence metric is to enable concepts to be extracted, disambiguating wellbeing from other concepts and showing instances of relative syntactical links. “This technique builds categories by grouping multiterm concepts (compound words) based on whether they contain words that are subsets or supersets of a word in the other” (IBM, 2018). Figure 4 displays these other concepts linked on their relative syntactical strength to “wellbeing”, mostly through phrases.

In summary, the analysis demonstrates the complexities of exploring “wellbeing” occurrence across the documents. To bring meaning to the text analysis, it is necessary to interpret the data in light of the context. A situated interpretation needs to be in reference to the political context in which the policy documents were created and informed by the theoretical stance of the interpreters. Thus, education policy can be conceptualized as a narrative (Prior et al., 2012) within political, social, economic and historical contexts. Building on the analysis of word weighting, co-occurrence and syntactical occurrence of wellbeing as a construct across policy documents, the next section will critically examine the narrative of wellbeing told across these documents.

## Discussion: wellbeing narrative across policy

Exploring how wellbeing emerges as a conceptualization across education policy documents presents a window of opportunity to interpret the ongoing proliferation of documents and initiatives around wellbeing. With the increasing urgency in shifting the policy focus from deficit outcomes to active promotion of wellbeing in education, we interpret the findings of the document analysis using a narrative (Roe, 1991) through appreciative and ecological lenses. As Ball (1993) suggested, “Much rests on the meaning, or the possible meanings, that we give to policy. It affects how we research and we interpret what we find” (p. 10). Tracing the complexities and contested notion of wellbeing in policy is challenging due to its multidimensionality. The following five-part narrative orients the discussion, outlines the challenges, draws implications, and makes recommendations.

### Orientation of wellbeing across the documents

Within the bicultural context of Aotearoa New Zealand, multiple and competing discourses about wellbeing exist across policy documents, adding to the complexity of wellbeing as a nebulous social and cultural mirage (Ereaut & Whiting, 2008). The predominant indigenous interpretation of wellbeing adopted in these policy documents was Durie’s (1994) Te Whare Tapa Whā, however, it only featured in three curricula and two system evaluation and improvement pieces. The remaining two-thirds of the documents were void of any definition, characterization or explanation of wellbeing, even if it was the document focus. Furthermore, the depth of wellbeing focus and explanation varied across documents, from nuanced references in the key competencies of the New Zealand Curriculum (Ministry of Education [MoE], 2007) to explicit explanations and examples in Health and Physical Education in the New Zealand Curriculum (MoE, 1999).

Within the education policy setting, groups of stakeholders are often the focus, for example, students; teachers; parents, caregivers, whānau; educational leaders; teacher educators; researchers and those in government and non-government educational agencies. These stakeholders can be considered as characters within the story woven into a policy narrative (Prior, 2014; Prior et al., 2012). Not all characters featured equally across the documents. As with other neoliberal-oriented education systems, children and young people as “students” were core characters in the policy narrative. This is not surprising with the focus on student outcomes and the child-centred origins of education in New Zealand (Middleton & May, 1997). Our analysis revealed that after the wellbeing of students, the next group of stakeholders was the wellbeing of leaders. It is important to note that the occurrence of leader wellbeing may have featured more strongly through the recent reforms in the professional leadership space (for instance, Education Council of Aotearoa New Zealand, 2018). In contrast, wellbeing featured less frequently in the documents in association with teachers and schools.

### Challenge

Teacher and school wellbeing is obscured across the policy documents that inform educational practices, which is salient given the apparent omission of teachers as policy actors (Ball, 2015). As Ball (1993) argues, “Nonetheless, policies are textual interventions into practice; and although many teachers (and others) are proactive, ‘writerly’ readers of texts, their readings and reactions are not constructed in circumstances of their own making.” (p. 12). To further complicate this apparent power imbalance, there are compounding issues likely to contribute to a lack of teacher wellbeing in the current educational climate within the national context. Recently, the Varkey Foundation Global Teacher Status Index researchers discovered that New Zealand teachers have the longest working hours of the 35 countries involved (Dolton et al., 2018). High levels of work-related stress, increasing hours, large workloads, substandard mentoring support and decreasing teacher morale were highlighted as core issues in New Zealand recent reports (Bonne & Wylie, 2017; New Zealand Council for Educational Research, 2018). Perhaps it is not surprising then that almost half of new secondary teachers abandon the profession within five years, contributing to the current teacher shortage (MoE, 2016, 2017a).

The absence of teacher wellbeing across the documents may have implications beyond the teachers themselves. First, Briner and Dewberry (2007) found a statistically significant causal relationship between educator wellbeing and student achievement. They also found that teacher wellbeing was associated with improved teacher health, presence and retention. Furthermore, educational institutions and communities benefit from increased educator wellbeing in terms of capacity to meet needs (Roffey, 2012).

The importance of “schools as health and wellbeing systems” (Boyd, 2009) is a critical factor in the promotion of wellbeing for all stakeholders; a challenge when centres, kura (Māori-medium schools) and school wellbeing does not feature strongly in the documents. A New Zealand government agency, the Education Review Office (ERO), advises early childhood service and schools on the care and education of students. They have written and published a series on wellbeing for success in which they use terms such as “a culture of wellbeing” to champion a schoolwide approach to wellbeing (Education Review Office [ERO], 2016). However, this represents one of the few places that school wellbeing features in policy documentation.

### Complication

A salient complication is the nebulous nature of wellbeing as a construct which is manifested in a lack of coherence across ecological strata. For instance, the text analysis confirmed the multidimensionality and complexity of wellbeing across the documents where the conceptualization of “wellbeing” or “well-being” varied significantly across the policy texts. Most documents were void of definition, characterization or description and, in fact, treated wellbeing as having a widely understood explanation.

To further complicate matters, the bicultural setting of Aotearoa New Zealand creates an imperative for all leaders, teachers, kura, centres and schools to interpret wellbeing in a way that honours Te Tiriti o Waitangi. Thus, a responsibility here is to see the possibilities of indigenous knowledge while attending to potential issues of appropriation through a decontextual and tokenistic interpretation of Māori constructs (McKinley, 2005). Additionally, schools, kura and centres have the ultimate task of interpreting the system-level curriculum for learners in their institutional setting.

To highlight the complexity of wellbeing, we draw on the example of the Intervention Triangle included in our document analysis. Introduced by the ERO (2013) as “a tool for identifying and prioritising school-wide and individual needs” (p. 15), the Intervention Triangle provides a frame to interpret the notion of wellbeing in a school context. The “Wellbeing for Success” series (ERO, 2013, 2015a, 2015b, 2016) draws on the work of Chafouleas et al. (2007) and The Collaborative for Academic, Social and Emotional Learning (CASEL). Both bodies of work portray tiers of intervention aimed at promoting student wellbeing within a school ecology.

Given the multiple internal and external factors, such as resourcing and legislative requirements, centres, kura and schools initially need to respond to a crisis or traumatic event. However, due to the small percentage of a student population with a crisis-level situation, the focus may shift to individual problems of vulnerable students, rather than schoolwide wellbeing promotion. In the supporting research, ERO (2016) argued: “schools that promoted wellbeing in their culture, curriculum and approaches were more able to respond to a traumatic event than schools that hadn’t promoted wellbeing” (p. 17). In “Responding to issues” (middle tier) there is still a clear pathway in place for seeking support from external agencies; whether or not the resourcing is available to respond to requests is a discussion for a different paper. “Promoting wellbeing” (top tier) has perhaps the greatest possibility for enhancing wellbeing for the student body. However, this is emergent and contingent on unpacking the complexities of wellbeing as defined by time and place.

In recent findings from a wellbeing-focused national survey of primary and intermediate schools, Boyd et al. (2016) describe the Promoting and Responding Triangle above as a decision-making tool where “prevention is better than cure” (p. 11). The authors contend that “building a proactive approach to promoting wellbeing and positive behaviour, that is aimed at all students and builds their competencies, will lead to fewer students needing extensive support” (p. 11). Rather than attending to the wellbeing and positive behaviour of individuals, they argue for the broader social context of behaviours to be considered. Boyd and colleagues elude that the intervention triangle may be interpreted by schools as a predominantly reactive model, rather than proactively promoting wellbeing in educational settings.

Consequently, attention to “Promoting wellbeing” for all students at all times may become a lower strategic, resourcing and programme priority than the other tiers. It is worth noting that this orientation may foster a focus on problem areas, rather than wellbeing promotion. Furthermore, the Promoting and Responding Triangle focuses on student wellbeing, presenting a single piece of the whole ecological system which needs to include teachers, leaders, whānau, institutions and communities. However, we have been struck by the triangle in terms of the possibilities of wellbeing as a transformative tool.

### Resolution

**Figure 5.**
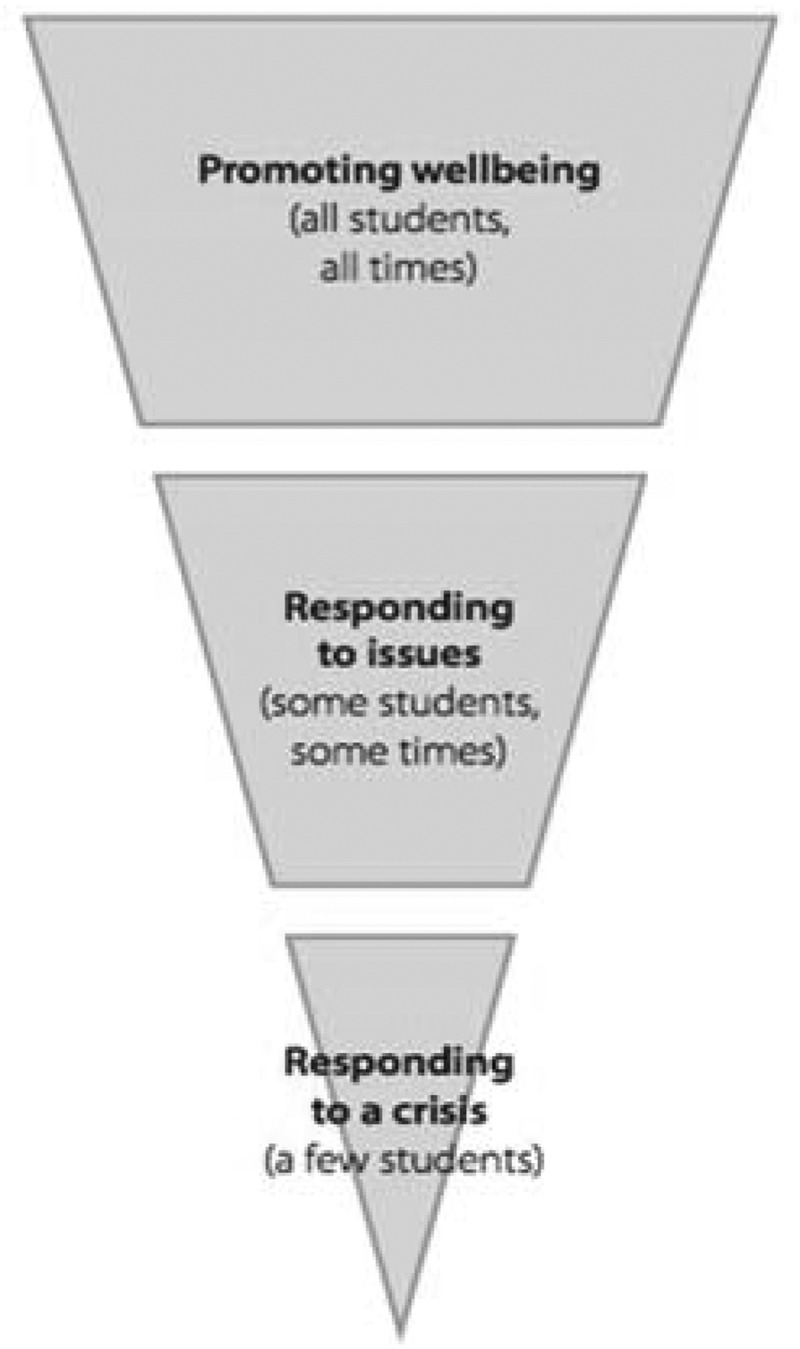
Promoting and Responding Triangle (ERO, 2016, p. 3)

Conceptualizing wellbeing as situated and contextualized within an ecological frame (Bronfenbrenner, 1979) enables an alternative perspective on the challenges and complications of enacting system-wide policy. An ecological lens provides insight into how the groups are highlighted in a stratified systems model, used in education to explore the interrelated strata within a wider ecological system (Bronfenbrenner, 1979). With an ecological lens, we can perceive the wellbeing of a system as holistically and biculturally contextualized with deliberate wellbeing practices across all strata. This aligns with Boyd et al. (2017), who found that “schools of all types have approaches in place that aim to promote all students’ wellbeing and belonging [see top tier of Figure 5 above], but these approaches may not be well embedded or part of a planned school-wide focus” (p. 60). We argue that the education community needs to recognize possibilities for deliberate and ecological wellbeing connections within Aotearoa New Zealand education policy and practice for the benefit of all stakeholders.

The predominant indigenous model, Te Whare Tapa Whā (Durie, 1994), opens up nuanced interpretations of wellbeing steeped within the richness of particular contexts. Furthermore, Te Whare Tapa Whā highlights the relational aspect of wellbeing, taha whānau. In the text analysis, the link strength of “practice” and “relationships” had the highest levels of co-occurrence with “wellbeing” across policy documents. Wellbeing as a relational practice was identified through the analysis, featuring in a dialectic sense that emphasizes that both groups and individuals need to be seen as a single entity “where each contains the other and cannot exist separately” (Alexakos, 2015, p. 15). Stepping beyond wellbeing promotion for stakeholders within ecological strata, some documents described a relational interdependence of wellbeing between and across strata. For example, wellbeing as a highly relational construct was found within *Te Whāriki*, the Ministry of Education, 2017a, which explicitly states “the wellbeing of each child is interdependent with the wellbeing of their kaiako (educators), parents and whānau (family)” (p. 20). A second example was *Tū Rangatira: Māori-medium educational leadership* (2010), where promoting wellbeing for learners, staff and whānau was at the core of effective leadership practice. This underscores the importance of the interdependence of wellbeing between all stakeholders.

**Figure 6.**
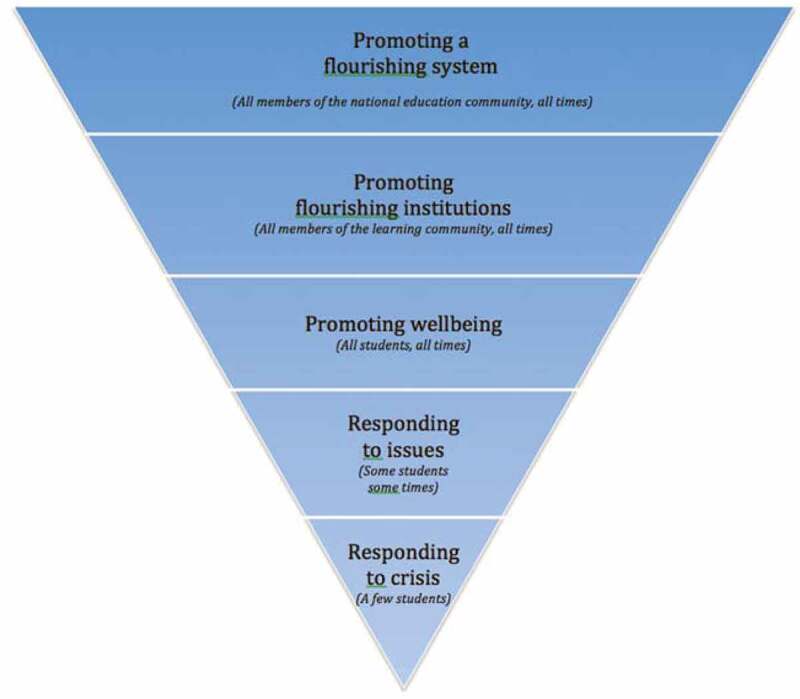
Flourishing transformation triangle

In considering the importance of wellbeing as contextualized and relational, we build on the Promoting and Responding Triangle (ERO, 2016) (see Figure 5). Our adaptation of the model shown above in Figure 6 was driven by possibilities of wellbeing as a transformative tool. The original model describes three tiers of action associated with promoting student wellbeing, as previously discussed. In order to deepen an appreciation of the complexities of wellbeing, we suggest two additional tiers are added to the triangle. Our penultimate tier is about “Promoting flourishing institutions” in which wellbeing is relevant to all members of the learning community at all times, which includes but is not limited to teachers, leaders, whānau (family), iwi, institutions and their wider communities. In recasting and embedding wellbeing as a transformative tool, our proposed ultimate tier, “Promoting a flourishing system”, acknowledges the intent of recent related government policy in Aotearoa New Zealand and beyond.

### Ending

According to Cooperrider et al. (2008), problem-based mindsets prompt limited constructs and outcomes in comparison to strengths-based or appreciative approaches, which serve to expand the potential scope of opportunities for particular contexts and purposes. Policy formation or policy drivers by definition are generally prompted as a political response to a problem or issue. Focusing on problems and resolutions contributes to Ball’s (2015) position that “policy works as a piecemeal of ‘fixing problems’” (p. 309). In seeking to disrupt the politicized “solving of problems”, we argue for a strengths-based approach that enables policy to be more than just *about* wellbeing but additionally *through* wellbeing.

To explore wellbeing as contextualized and strengths-based, Table II presents the different permutations summarized using a Johari window (Luft & Ingham, 1955). Although the column and row headings suggest a closed system, the intention is to highlight the points of intersection between focus and context. The first row presents traditional approaches to problem-based policy and practice. In quadrant A, problem-based decontextualized policy is likely to have a unidimensional focus on a specific systemic issue of concern that has prompted political action. For example, anti-bullying programmes adopted from other jurisdictions have been critiqued as international policy borrowing (Steiner-Khamsi, 2006). In cases where the focus remains on problems, albeit that these are contextualized (see quadrant B), centre, kura, school or community action is likely to be contained within a narrative of ill-being, for instance, responses to student mental health challenges within a specific setting. Where strengths-based approaches to a wellbeing policy and practice are decontextualized, the outcomes tend to be generalized applications of wellbeing. This is evident in the prolific calls in the media for ways to enhance wellbeing in popular culture and within the education sector, critiqued by some commentators as the commodification of wellbeing (Forbes, 2016 (see quadrant C). As explained in the resolution section, taking an ecological and bicultural interpretation of a contextualized strength-based approach points to wellbeing policy and practice, which deliberately encompasses all tiers of a holistically contextualized system, as in Aotearoa New Zealand (see quadrant D). See Table 1 for the set of documents for analysis.

**Table 1.** The document set for analysis

The document set for analysis		Intended function of documents
1	MoE (1999). *Health and Physical Education in the New Zealand Curriculum*. Learning Media.	Curricula
2	MoE (2007). *The New Zealand Curriculum*. Learning Media.	Curricula
3	MoE (2008). *Kiwi Leadership for Principals: Principals as Educational Leaders*. Ministry of Education.	Leadership guidelines
4	MoE (2010). *Tū Rangatira: Maori Medium Educational Leadership*. Huia Publishers.	Leadership guidelines
5	MoE (2012). *Leading from the Middle: Educational Leadership for Middle and Senior Leaders*. Learning Media.	Leadership guidelines
6	ERO (2013). *Wellbeing for Success: Draft Evaluation Indicators for Student Wellbeing*.	System evaluation and improvement
7	ERO (2015a). *Wellbeing for Young People’s Success at Primary School*.	System evaluation and improvement
8	ERO (2015b). *Wellbeing for Young People’s Success at Secondary School*.	System evaluation and improvement
9	ERO (2016). *Wellbeing for Success: A resource for schools.*	System evaluation and improvement
10	Ministry of Education (2016). *Health and wellbeing programmes.*	Legal and regulatory frameworks
11	Education Council of Aotearoa New Zealand (2017). *Our Code Our Standards: Code of Professional Responsibility for the Teaching Profession*.	Professional standards
12	MoE (2017a). *Te Whāriki: He whāriki mātauranga mō ngā mokopuna o Aotearoa: Early Childhood Curriculum*. Ministry of Education.	Curricula
13	MoE (2017b). *National Administration Guidelines* (NAGs).	Legal and regulatory frameworks
14	Education Council of Aotearoa New Zealand (2018). *The Leadership Strategy for the teaching profession of Aotearoa New Zealand: Enabling every teacher to develop their leadership capability*. Education Council.	Professional standards
15	Education Council of Aotearoa New Zealand (2018). *Educational Leadership Capability Framework*. Education Council.	Professional standards

**Table II.** Conceptualization and contextualization of wellbeing

Focus/Purpose	Decontextualized	Contextualized
Problem-based	(A) Unidimensional specific issue	(B) Situation-based responses to specific issues
Strengths-based	(C) Generalized and commodified	(D) Multidimensional, situated and deliberate

On reflection, various strengths and limitations arose through the process of document analysis. The application of the text mining tool across the documents enabled comprehensive mapping of concept frequency and relationship strength. However, the strength of document analysis is dependent on the availability and quality of documents. Further, Prior (2014) pointed out that documents are necessarily situated in a context bounded by time and place, contributing to the complexity of document analysis as a research approach (Hard et al., 2018). Relevant to the Aotearoa New Zealand bicultural context, IBM SPSS Modeller programme (2018), as with other text mining programmes, did not include Te Reo Māori as a language to use for text analysis. To minimize this issue, Te Reo Māori words were manually grouped alongside the English concepts. For instance, “tamariki’’ was manually grouped with “children”. Overall, the document analysis provided a mechanism for the development of a narrative form, which provided nuanced insights into wellbeing in education policy.

## Concluding comments

This paper sought to explore how public policy can present wellbeing so that the interrelatedness, complexity, and contextual nature of wellbeing is reflected in educational settings. Wellbeing has been politicized as a social and cultural mirage, making it challenging to contextualize and reflect its interrelated, complex, and relational characteristics. The document analysis revealed weak associations of wellbeing with teachers and schools. Findings highlighted that current public education policy in Aotearoa New Zealand does not interpret wellbeing as interrelated, complex or contextual. Our wellbeing narrative generated from the document analysis underscores the need to recognize possibilities for deliberate and ecological wellbeing connections within our bicultural context for the benefit of all stakeholders. Furthermore, strengths-based, contextualized approaches to wellbeing policy and embedded practices need to reach from specific to multidimensional manifestations and incorporate multiple and competing discourses.

To shift the focus on wellbeing in educational settings, we argue that the narrow focus on individual student wellbeing needs to be reframed to promote a holistic, ecological perspective encompassing the interrelatedness of wellbeing within the education sector of Aotearoa New Zealand and possibly beyond. Within a strengths-based and contextualized approach lies the potential for wellbeing policy and practice to be emergent and contingent in response to the ever-evolving context. Our presented models provide opportunities for reflection and promotion of wellbeing to provide a relational, contextualized strengths-based approach to policy and practice. Understanding the nature of wellbeing as multidimensional and complex provides a policy window to generate a strengths-based policy orientation to promote wellbeing in education settings. In regards to wellbeing, we acknowledge the challenges and timeliness of generating forms of institutional transformation (Ball, 2015). Wellbeing continues to be on the global agenda. Future wellbeing policy in Aotearoa New Zealand needs to be formed and enacted from a contextualized strengths-based approach that promotes wellbeing as multidimensional, situated and deliberate for all stakeholders.
